# Public Opinion on Priorities Toward Fair Allocation of Ventilators During COVID-19 Pandemic: A Nationwide Survey

**DOI:** 10.3389/fpubh.2021.753048

**Published:** 2021-12-14

**Authors:** Mohsen Abbasi-Kangevari, Shahnam Arshi, Hossein Hassanian-Moghaddam, Ali-Asghar Kolahi

**Affiliations:** Social Determinants of Health Research Center, Shahid Beheshti University of Medical Sciences, Tehran, Iran

**Keywords:** coronavirus infections, health care rationing, ethics, health policy, resource allocation, SARS-CoV-2, mechanical ventilators

## Abstract

**Background:** The rapidly growing imbalance between supply and demand for ventilators during the COVID-19 pandemic has highlighted the principles for fair allocation of scarce resources. Failing to address public views and concerns on the subject could fuel distrust. The objective of this study was to determine the priorities of the Iranian public toward the fair allocation of ventilators during the COVID-19 pandemic.

**Methods:** This anonymous community-based national study was conducted from May 28 to Aug 20, 2020, in Iran. Data were collected via the Google Forms platform, using an online self-administrative questionnaire. The questionnaire assessed participants' assigned prioritization scores for ventilators based on medical and non-medical criteria. To quantify participants' responses on prioritizing ventilator allocation among sub-groups of patients with COVID-19 who need mechanical ventilation scores ranging from −2, very low priority, to +2, very high priority were assigned to each response.

**Results:** Responses of 2,043 participants, 1,189 women, and 1,012 men, were analyzed. The mean (SD) age was 31.1 (9.5), being 32.1 (9.3) among women, and 29.9 (9.6) among men. Among all participants, 274 (13.4%) were healthcare workers. The median of assigned priority score was zero (equal) for gender, age 41–80, nationality, religion, socioeconomic, high-profile governmental position, high-profile occupation, being celebrities, employment status, smoking status, drug abuse, end-stage status, and obesity. The median assigned priority score was +2 (very high priority) for pregnancy, and having <2 years old children. The median assigned priority score was +1 (high priority) for physicians and nurses of patients with COVID-19, patients with nobel research position, those aged <40 years, those with underlying disease, immunocompromise status, and malignancy. Age>80 was the only factor participants assigned −1 (low priority) to.

**Conclusions:** Participants stated that socioeconomic factors, except for age>80, should not be involved in prioritizing mechanical ventilators at the time of resources scarcity. Front-line physicians and nurses of COVID-19 patients, pregnant mothers, mothers who had children under 2 years old were given high priority.

## Introduction

The coronavirus disease (COVID-19) pandemic continues to place extraordinary demands on healthcare systems and has resulted in severe shortages of essential resources and services (1). In the pandemic's early days, the face masks' shortages became increasingly concerning (2). Nevertheless, among all the medical resources, scarcity of ventilators could be the most challenging, as there is typically limited time if mechanical ventilation is essential (3). Another limiting factor is the availability of trained healthcare professionals to operate ventilators safely, especially considering the catastrophic casualty of COVID-19 of healthcare professionals (4).

The rapidly growing imbalance between supply and demand for ventilators has raised the question of how to allocate them during the COVID-19 pandemic fairly. Research has been ongoing to investigate the main principles for allocating scarce medical resources during pandemics (5–7). In this sense, physicians should not be faced with situations where they must decide which patient to treat due to the risk of human error and the life-long emotional toll (8). Therefore, prioritization recommendations and guidelines have been developed in the hope of helping physicians, especially those less experienced, with the real-time decision-making process based on the resources and contexts (1, 9).

The proposed principles for resource allocation could become controversial in the eyes of the public. Even the seemingly most holistic approaches for resource allocation proposed by healthcare systems could result in inequalities among patients (10). This could raise serious concerns about their fairness and result in loss of public trust in health care systems. Therefore, people need to be involved in developing and evaluating such policies as stakeholders. Failing to address public views and concerns could fuel distrust and negatively affect compliance to health-promoting measures (11).

The objective of this study was to determine the priorities of the Iranian public toward the fair allocation of ventilators during the COVID-19 pandemic via an online survey.

## Methods

This community-based national study was approved by the Ethical Committee of Shahid Beheshti University of Medical Sciences under code IR.SBMU.RETECH.REC.1399.167. Participation was anonymous and upon the participant's own decision.

### Setting and Sampling

This anonymous network-sampling survey was conducted from May 28 to Aug 20, 2020, in Iran. Data were collected via the Google Forms platform, using an online self-administrative questionnaire. An invitation post with a link to the questionnaire was circulated online on popular social networks in Iran, including Telegram, Instagram, WhatsApp, Twitter, and LinkedIn. Participants were Iranian adults currently living in Iran who agreed to participate in the study.

### Variables and the Questionnaire

Variables included socio-demographic characteristics and the criteria for prioritizing the ventilators during the pandemic. Socio-demographic characteristics included participants' age, sex, ethnicity, religion, literacy, the province of residence in Iran, being a healthcare professional, marital status, number of alive children, smoking status, having underlying diseases, previous history of COVID-19, being tested for COVID-19, being admitted for COVID-19, and knowing someone with COVID-19.

The criteria for prioritizing the ventilators during the pandemic included age, nationality, religion, occupation, socioeconomic status, smoking status, drug abuse, and underlying diseases.

A panel of ten experts, including two public health experts, two anesthesiologists, two emergency medicine experts, two pulmonologists, and two infectious diseases specialists, evaluated the questionnaire's content validity. An item discrimination analysis was conducted for each scale to eliminate too tricky or easy items. Factor analysis was performed for factor structure. Separate test-retest over 2 weeks were held for the three scales of the questionnaire. The test-retest correlation was 0.91; Kuder-Richardson-20 was used to prevent internal consistency overestimation; the coefficient was 0.87. The pilot survey was conducted on twenty men, and twenty women recruited online via convenience sampling method.

### Data Analysis

To quantify participants' responses on prioritizing ventilator allocation among sub-groups of patients with COVID-19 who need mechanical ventilation scores were assigned to each response: “very low priority” was considered as “−2,” “low priority” was considered as “−1,” “equal priority” was considered as “0,” “high priority” was considered as “+1,” and “very high priority” was considered as “+2.” To measure the distribution of responses, mean, standard deviation (SD), 95% confidence interval (95% CI), median, interquartile range (IQR), mode, and skewness were reported. Categorical variables were analyzed by the Chi-Square test. For analyzing the differences among means of two groups and three groups or more, independent-sample *t*-test and one-way analysis of variance (ANOVA) test were used. Statistical analyses were performed using IBM SPSS Statistics 21. A probability level of <0.05 was considered significant.

## Results

Responses of 2,043 participants were analyzed. There were no missing values. The mean (SD) age was 31.1 (9.5) [range = 18–80, being 32.1 (9.3) among women, and 29.9 (9.6) among men]. Among participants, 259 (12.7) smoked. Other socio-demographic characteristics are presented in Table 1.

**Table 1:** Socio-demographic characteristics of participants.

**Variable**	***N*** **(%)**
**Sex**	
Female	1,122 (54.9)
Male	921 (45.1)
**Marital status**	
Never married	823 (40.2)
Engaged	45 (2.2)
Married	1,127 (55.2)
Divorced	40 (2.0)
Widowed	8 (0.4)
**Ethnicity**	
Fars	1,145 (56.1)
Turk/Azari	449 (22.0)
Kurd	129 (6.3)
Lor	88 (4.3)
Other	232 (11.3)
**Literacy**	
High school diploma	475 (23.2)
Associate degree	121 (5.9)
Bachelor	861 (42.1)
Master	375 (18.4)
Ph.D. and higher	197 (9.8)
Other	14 (5.6)
**Healthcare worker**	
Yes	274 (13.4)
No	1,769 (86.6)

Among all participants, 151 (6.9%) said they had a history of COVID-19, 268 (12.2%) said they smoked, 332 (15.1%) reported having underlying diseases, 13 (0.6%) reported being intubated due to COVID-19, and 899 (40.8) said they knew someone who had been admitted due to COVID-19.

The majority of participants believed that socioeconomic determinants including gender, age below 80, nationality, religion, and employment should not be involved in prioritizing mechanical ventilators at the time of resource scarcity. Participants also did not consider smoking and drug abuse to be determinants of prioritization. Nevertheless, participants responded that patients aged above 80 receive very low priority. Front-line physicians and nurses of COVID-19 patients were given very high priority, along with pregnant mothers and those who had children under 2 years old. While end-stage status and obesity were considered unimportant in resource allocation, having underlying diseases, malignancy, and immunocompromised status were given high priority (Tables 2, 3).

**Table 2:** Participants' responses on prioritizing ventilator allocation among sub-groups of patients with COVID-19 who need mechanical ventilation.

**Factor**	**Very low priority (n%)**	**Low priority (n%)**	**Equal priority (n%)**	**High priority (n%)**	**Very high priority (n%)**
Female gender	12 (0.6)	8 (0.4)	1,515 (74.2)	269 (13.2)	239 (11.6)
**Age (years)**					
<40	14 (0.7)	99 (4.8)	870 (42.6)	538 (26.3)	522 (25.6)
41–60	15 (0.8)	74 (3.6)	936 (45.8)	756 (37.0)	262 (12.8)
61–80	123 (6.0)	483 (23.6)	801 (39.2)	287 (14.1)	349 (17.1)
>80	585 (28.6)	463 (22.7)	534 (26.1)	164 (8.0)	297 (14.6)
Iranian nationality	26 (1.3)	30 (1.5)	1,300 (63.6)	213 (10.4)	474 (23.2)
Muslim religion	61 (3.0)	13 (0.6)	1,792 (87.7)	64 (3.1)	113 (5.6)
**Physician profession**					
of COVID-19 patients	10 (0.5)	6 (0.3)	509 (24.9)	578 (28.3)	940 (46.0)
not for COVID-19 patients	26 (1.3)	30 (1.5)	1,388 (67.9)	427 (20.9)	172 (8.4)
**Nurse profession**					
of COVID-19 patients	9 (0.4)	8 (0.4)	490 (24.0)	651 (31.9)	885 (43.3)
not for COVID-19 patients	25 (1.2)	26 (1.3)	1,404 (68.7)	425 (20.8)	163 (8.0)
Other healthcare professionals	27 (1.3)	23 (1.1)	1,535 (75.1)	324 (15.9)	134 (6.6)
High socioeconomic status	142 (7.0)	86 (4.2)	1,743 (85.3)	43 (2.1)	29 (1.4)
High-profile governmental position	637 (31.2)	124 (6.1)	1,182 (57.9)	62 (3.0)	38 (1.8)
Nobel research position	Zero	Zero	939 (42.6)	631 (28.7)	631 (28.7)
High-profile occupation	77 (3.8)	39 (1.9)	1,560 (76.3)	274 (13.4)	93 (4.6)
Celebrities	87 (4.3)	46 (2.3)	1,735 (84.9)	123 (6.0)	52 (2.5)
Unemployment	24 (1.2)	24 (1.2)	1,799 (88.1)	97 (4.7)	99 (4.8)
Pregnancy	9 (0.4)	3 (0.1)	198 (9.7)	651 (31.9)	1182 (57.9)
Having <2 years old child	7 (0.3)	Zero	274 (13.4)	655 (32.1)	1107 (54.2)
Smoking	114 (5.6)	212 (10.4)	1,517 (74.3)	116 (5.7)	84 (4.0)
Drug abuse	332 (16.3)	390 (19.0)	1,209 (59.2)	73 (3.6)	39 (1.9)
Having underlying disease	15 (0.7)	54 (2.6)	564 (27.6)	815 (39.9)	595 (29.2)
Immunocompromise	30 (1.5)	70 (3.4)	509 (24.9)	753 (36.9)	681 (33.3)
Malignancy	78 (3.8)	172 (8.4)	520 (25.5)	639 (31.3)	634 (31.0)
End-stage status	428 (20.9)	510 (25.0)	802 (39.3)	137 (6.7)	166 (8.1)
Obesity	16 (0.8)	43 (2.1)	1,649 (80.7)	210 (10.3)	125 (6.1)

**Table 3:** Dispersion measures of participants' responses on prioritizing ventilator allocation among sub-groups of patients with COVID-19 who need mechanical ventilation.

**Factor**	**Mode**	**Mean (SD)**	**95% CI**	**Median (IQR)**	**Skewness**
Female gender	Equal	0.4 (0.7)	0.3 to 0.4	0 (0 to 1)	1.2
**Age (years)**					
<40	Equal	0.7 (0.9)	0.7 to 0.8	1 (0,2)	Zero
41–60	Equal	0.6 (0.8)	0.5 to 0.6	0 (0,1)	0.1
61–80	Equal	0.1 (1.2)	0.1 to 0.2	0 (−1,1)	0.2
>80	Very low	−0.4 (1.4)	(−0.5) to (−0.3)	−1 (−2,0)	0.5
Iranian nationality	Equal	0.5 (0.9)	0.5 to 0.6	0 (0,1)	0.5
Muslim religion	Equal	0.1 (0.6)	0.06 to 0.11	0 (0,0)	0.7
**Physician profession**					
of COVID-19 patients	Very high	1.2 (0.9)	1.1 to 1.2	1 (0,2)	−0.6
not for COVID-19 patients	Equal	0.3 (0.7)	0.3 to 0.4	0 (0,1)	0.6
**Nurse profession**					
of COVID-19 patients	Very high	1.2 (0.8)	1.1 to 1.2	1 (1,2)	−0.6
not for COVID-19 patients	Equal	0.3 (0.7)	0.3 to 0.4	0 (0,1)	0.7
Other healthcare professionals	Equal	0.3 (0.7)	0.2 to 0.3	0 (0,0)	0.8
High socioeconomic status	Equal	−0.1 (0.6)	(−0.2) to (−0.1)	0 (0,0)	−1.2
High-profile governmental position	Equal	−0.6 (1)	(−0.6) to (−0.5)	0 (−2,0)	−0.2
Nobel research position	Equal	0.9 (0.8)	0.8 to 0.9	1 (0,2)	0.3
High-profile occupation	Equal	0.1 (0.7)	0.1 to 0.2	0 (0,0)	Zero
Celebrities	Equal	0 (0.6)	−0.01 to 0.04	0 (0,0)	−0.4
Unemployment	Equal	0.1 (0.5)	0.08 to 0.13	0 (0,0)	1.4
Pregnancy	Very high	1.5 (0.7)	1.4 to 1.5	2 (1,2)	−1.3
Having <2 years old child	Very high	1.4 (0.7)	1.3 to 1.4	2 (1,2)	−1
Smoking	Equal	−0.1 (0.7)	(−0.1) to 0	0 (0,0)	−0.1
Drug abuse	Equal	−0.4 (0.9)	(−0.5) to (−0.4)	0 (−1,0)	−0.3
Having underlying disease	High	0.9 (0.9)	0.9 to 1	1 (0,2)	−0.5
Immunocompromise	High	1 (0.9)	0.9 to 1	1 (0,2)	−0.6
Malignancy	High	0.8 (1.1)	0.7 to 0.8	1 (0,2)	−0.6
End-stage status	Equal	−0.4 (1.1)	(−0.5) to (−0.4)	0 (−1,0)	0.4
Obesity	Equal	0.2 (0.6)	0.17 to 0.22	0 (0,0)	1.3

Compared with men, women assigned higher scores to the female gender, age 41–60, having underlying disease, being immunocompromised, having malignancy, or having an end-stage disease. In addition, they assigned lower scores to Iranian nationality compared with men (Table 4).

**Table 4:** The mean priority score assigned to each sub-group of patients with COVID-19 among men and women.

**Factor**	**Women**		**Men**		***p***
	**(***n*** = 1,189)**		**(***n*** = 1,012)**		
	**Mean (SD)**	**95% CI**	**Mean (SD)**	**95% CI**	
Female gender	0.4 (0.7)	0.4 to 0.4	0.3 (0.7)	0.2 to 0.3	<0.001
**Age (years)**					
<40	0.8 (0.9)	0.7 to 0.8	0.6 (0.9)	0.6 to 0.7	N/S^*^
41–60	0.6 (0.8)	0.6 to 0.7	0.5 (0.8)	0.4 to 0.5	<0.001
61–80	0.2 (1.1)	0.1 to 0.2	0.1 (1.2)	0 to 0.2	N/S
>80	−0.4 (1.3)	(−0.5) to (−0.3)	−0.4 (1.5)	(−0.5) to (−0.3)	N/S
Iranian nationality	0.5 (0.9)	0.4 to 0.5	0.6 (0.9)	0.5 to 0.7	<0.001
Muslim religion	0.1 (0.5)	0.1 to 0.1	0.1 (0.7)	0 to 0.1	N/S
**Physician profession**					
of COVID-19 patients	1.2 (0.8)	1.1 to 1.2	1.2 (0.9)	1.2 to 1.3	N/S
not for COVID-19 patients	0.4 (0.7)	0.3 to 0.4	0.3 (0.8)	0.3 to 0.4	N/S
**Nurse profession**					
of COVID-19 patients	1.1 (0.8)	1.1 to 1.2	1.2 (0.9)	1.1 to 1.2	N/S
not for COVID-19 patients	0.3 (0.7)	0.3 to 0.4	0.3 (0.7)	0.3 to 0.4	N/S
Other healthcare professionals	0.3 (0.6)	0.3 to 0.3	0.2 (0.7)	0.2 to 0.3	0.004
High socioeconomic status	−0.1 (0.5)	(−0.1) to (−0.1)	−0.2 (0.7)	(−0.2) to (−0.1)	N/S
High-profile governmental position	−0.5 (0.9)	(−0.6) to (−0.5)	−0.7 (1.1)	(−0.7) to (−0.6)	0.04
Nobel research position	0.8 (0.8)	0.8 to 0.9	0.9 (0.8)	0.9 to 1	<0.001
High-profile occupation	0.1 (0.6)	0.1 to 0.2	0.1 (0.8)	0.1 to 0.2	N/S
Celebrities	0.1 (0.5)	0 to 0.1	0 (0.7)	(−0.1) to 0	0.01
Unemployment	0.1 (0.5)	0.1 to 0.1	0.1 (0.6)	0.1 to 0.1	N/S
Pregnancy	1.5 (0.7)	1.4 to 1.5	1.5 (0.7)	1.4 to 1.5	N/S
Having <2 years old child	1.4 (0.7)	1.4 to 1.5	1.3 (0.8)	1.3 to 1.4	<0.001
Smoking	0 (0.7)	(−0.1) to 0	−0.1 (0.8)	(−0.2) to (−0.1)	<0.001
Drug abuse	−0.4 (0.9)	(−0.5) to (−0.4)	−0.5 (0.9)	(−0.5) to (−0.4)	0.02
Having underlying disease	1 (0.8)	1 to 1.1	0.8 (0.9)	0.8 to 0.9	<0.001
Immunocompromise	1.1 (0.9)	1 to 1.1	0.8 (1)	0.8 to 0.9	<0.001
Malignancy	0.9 (1)	0.9 to 1	0.6 (1.1)	0.5 to 0.7	<0.001
End-stage status	−0.3 (1.1)	(−0.4) to (−0.3)	−0.6 (1.2)	(−0.6) to (−0.5)	<0.001
Obesity	0.2 (0.6)	0.2 to 0.3	0.1 (0.6)	0.1 to 0.2	<0.001

* *Not significant*.

Among all participants, 274 (13.4%) were healthcare workers. Most healthcare workers believed that socioeconomic determinants, including gender, age below 80, nationality, religion, and employment, should not determine ventilator prioritization. They also did not consider smoking and drug abuse to be determinants of prioritization. Although they said that patients with underlying diseases, immunocompromise, or malignancy should receive the same priority as others, they assigned very low scores to patients with end-stage disease.

Compared with non-healthcare workers, healthcare workers assigned higher scores to age <40, healthcare workers, and Nobel researchers. They also assigned lower scores to having underlying disease, being immunocompromised, having malignancy, or having an end-stage disease (Table 5).

**Table 5:** The mean priority score assigned to each sub-group of patients with COVID-19 among healthcare workers and non-healthcare workers.

**Factor**	**Healthcare workers**		**Non-healthcare workers**		***p***
	**(***n*** = 274)**		**(***n*** = 1,769)**		
	**Mean (SD)**	**95% CI**	**Mean (SD)**	**95% CI**	
Female gender	0.3 (0.6)	0.2 to 0.4	0.4 (0.7)	0.3 to 0.4	N/S
**Age (years)**					
<40	0.8 (0.9)	0.7 to 0.9	0.7 (0.9)	0.7 to 0.7	0.02
41–60	0.6 (0.8)	0.6 to 0.7	0.6 (0.8)	0.5 to 0.6	N/S
61–80	0.1 (1)	(−0.1) to 0.2	0.1 (1.2)	0.1 to 0.2	N/S
>80	−0.6 (1.2)	(−0.7) to (−0.5)	−0.4 (1.4)	(−0.4) to (−0.3)	0.01
Iranian nationality	0.5 (0.8)	0.4 to 0.6	0.5 (0.9)	0.5 to 0.6	N/S
Muslim religion	0 (0.5)	0 to 0.1	0.1 (0.6)	0.1 to 0.1	N/S
**Physician profession**					
of COVID-19 patients	1.3 (0.8)	1.2 to 1.4	1.2 (0.9)	1.1 to 1.2	N/S
not for COVID-19 patients	0.6 (0.8)	0.6 to 0.7	0.3 (0.7)	0.3 to 0.3	<0.001
**Nurse profession**					
of COVID-19 patients	1.2 (0.8)	1.1 to 1.3	1.2 (0.8)	1.1 to 1.2	N/S
not for COVID-19 patients	0.6 (0.8)	0.5 to 0.7	0.3 (0.7)	0.3 to 0.3	<0.001
Other healthcare professionals	0.5 (0.7)	0.4 to 0.6	0.2 (0.6)	0.2 to 0.2	<0.001
High socioeconomic status	0 (0.6)	(−0.1) to 0	−0.1 (0.6)	(−0.2) to (−0.1)	<0.001
High-profile governmental position	−0.5 (1)	(−0.6) to (−0.4)	−0.6 (1)	(−0.7) to (−0.6)	N/S
Nobel research position	1.1 (0.9)	1 to 1.2	0.8 (0.8)	0.8 to 0.9	<0.001
High-profile occupation	0.3 (0.7)	0.2 to 0.3	0.1 (0.7)	0.1 to 0.2	<0.001
Celebrities	0.2 (0.7)	0.1 to 0.3	0 (0.6)	0 to 0	<0.001
Unemployment	0.1 (0.5)	0 to 0.2	0.1 (0.5)	0.1 to 0.1	N/S
Pregnancy	1.4 (0.7)	1.3 to 1.5	1.5 (0.7)	1.4 to 1.5	N/S
Having <2 years old child	1.3 (0.8)	1.2 to 1.4	1.4 (0.7)	1.4 to 1.4	0.02
Smoking	−0.1 (0.7)	(−0.2) to 0	−0.1 (0.7)	(−0.1) to 0	N/S
Drug abuse	−0.5 (0.9)	(−0.6) to (−0.4)	−0.4 (0.9)	(−0.5) to (−0.4)	N/S
Having underlying disease	0.8 (0.9)	0.7 to 0.9	1 (0.9)	0.9 to 1	<0.001
Immunocompromise	0.8 (1)	0.7 to 0.9	1 (0.9)	0.9 to 1	<0.001
Malignancy	0.4 (1.2)	0.3 to 0.6	0.8 (1.1)	0.8 to 0.9	<0.001
End-stage status	−0.7 (1.1)	(−0.9) to (−0.6)	−0.4 (1.1)	(−0.4) to (−0.3)	<0.001
Obesity	0.2 (0.7)	0.1 to 0.3	0.2 (0.6)	0.2 to 0.2	N/S

^*^Not significant.

No correlations were observed with participants' responses on resources allocation and their age, ethnicity, religion, literacy, the province of residence in Iran, marital status, number of alive children, smoking status, having underlying diseases, previous history of COVID-19, being tested for COVID-19, being admitted for COVID-19, and knowing someone with COVID-19.

## Discussion

The study showed that most participants believed that socioeconomic factors, including gender, age below 80, nationality, religion, employment; smoking and drug abuse; medical conditions including end-stage status, and obesity, should not be involved in prioritizing mechanical ventilators at the time of resources scarcity. Front-line physicians and nurses of COVID-19 patients, pregnant mothers, mothers who had children under 2 years old, patients with underlying diseases, malignancy, or immunocompromised status were given high priority. On the contrary, participants assigned age above 80 very low priority.

Participants did not consider age <80 to be a deciding factor in resource allocation. However, a study on the general public in the US reported that participants favored allocating more ventilators to patients of younger age groups (10). This conforms with the current proposed guidelines that prioritize younger patients to receive scarce medical resources (9, 12, 13). Nevertheless, there are controversies in the literature and ethical guidelines regarding using age as a screening factor for resource allocation (14). Although higher age groups are associated with higher mortality rates due to COVID-19 (15, 16), poor outcomes among the elderly could be attributable to comorbidities with a higher prevalence among older age groups (16, 17). Thus, some studies argue that age should only be used as a tiebreaker criterion among patients with similar severity of COVID-19, not the only criterion to determine screening decisions (18–20). In this context, it is the duty of health authorities and the media not to disseminate fear among the older age groups and ease their concerns via effective communication.

The majority of participants agreed that ventilators should not be allocated based on non-clinical irrelevant aspects. They did not consider gender, employment, financial condition, or social relations as deciding factors. Moreover, almost 90% of participants said that religion should not be considered a prioritization factor, which is satisfactory given that crises like the COVID-19 pandemic tend to fuel conflicts. Some two-thirds of participants considered patients of other nationalities to have equal priority as Iranians. Iran is host to millions of refugees from neighboring countries, mostly Afghanistan. Given the vulnerable state of refugees in terms of health and care-seeking behavior (21), policies need to be directed to avoid the stigmatization against refugees in resource allocation. Ventilators should not be allocated based on morally irrelevant aspects. In this sense, all stakeholders need to bear in mind that the principles of accessibility, dignity, and equal opportunities need to be considered in allocating scarce resources.

Participants acknowledged the consideration of the patients' instrumental value. They assigned higher scores to physicians and nurses treating COVID-19 patients, as well as high-profile researchers. Those supporting the role of instrumental value argue that the prioritization of access to the resources for patients with essential skills to save others' lives could potentially multiply the net benefit to society (12, 22). Participants also assigned higher scores to pregnant mothers or those who had children under 2 years old. Some triage models consider having someone dependent on care as a criterion to lessen the harm caused to families and society (12).

Participants would prioritize patients with underlying diseases to access ventilators. Moreover, they would equally allocate ventilators to patients with end-stage status. On the contrary, healthcare workers assigned very low scores to patients with end-stage status. This calls for more effective communication and knowledge translation by public health authorities and the media to regularly convey the prognostic factors of COVID-19 based on emerging evidence to justify people's expectations from the healthcare systems. The disruption of the resource supply imposed by the COVID-19 pandemic has challenged the public's shared belief that healthcare services are provided whenever requested. Thus, people need to beware of the catastrophic aftermaths of not abiding by preventive protocols (23, 24). To date, global organizations have proposed no unique criteria for the fair allocation of mechanical ventilators. Such protocols need to be developed and implemented regarding each country's local context or state (25). As the COVID-19 pandemic is a rapidly evolving crisis, it is of paramount importance to regularly reevaluate current practices based on the emerging evidence and feedback of all stakeholders, including public health authorities, decision-makers, clinicians the general public.

## Strengths and Limitations

This is among the few studies to assess the public opinions on priorities toward fair allocation of mechanical ventilators during the COVID-19 pandemic. Findings could empower public health authorities better to understand people's views on the matter as stakeholders to avoid public distrust and improve people's compliance to health-promoting measures. Nevertheless, the study does not overshadow the need for accelerated production and enhanced distribution of ventilators (26). Operational management aspects of allocating ventilators also need to be taken into account at all levels to enhance prompt response to the devastating demands as imposed by the COVID-19 pandemic (27).

Some limitations must be acknowledged. The study was conducted via Google Forms, an online survey platform because there was no representative online platform for rapid surveys among people in Iran. While the study could be subject to selection bias, its rapid conduction via an online platform could outweigh its limitation. Compared to Iran's most recent national population statistics, our sample was over-representative of women and health workers. Considering that healthcare workers also comprise most of the authorities of the healthcare system in Iran, their opinions were compared with the public to gain a deeper understanding of the potential differences in their points of view. Although the Internet penetration rate in Iran is high, the elderly and vulnerable groups in rural areas might not have access to the platform, who might have gotten underrepresented in the study. Participants were asked to read the questionnaire to their parents and grandparents to ensure higher participation of those groups; however, such voluntary measures are not guaranteed. Some 99.6% of Iranians are Muslims (28), thus religion had no bearing on the choice of allocation in this study.

## Conclusion

Participants stated that socioeconomic factors, except for age>80, should not be involved in prioritizing mechanical ventilators at the time of resources scarcity. Front-line physicians and nurses of COVID-19 patients, pregnant mothers, mothers who had children under 2 years old were given high priority.

## Data Availability Statement

The raw data supporting the conclusions of this article will be made available by the authors, without undue reservation.

## Ethics Statement

The studies involving human participants were reviewed and approved by Ethical Committee of Shahid Beheshti University of Medical Sciences, Tehran, Iran under code IR.SBMU.RETECH.REC.1399.167. The patients/participants provided their written informed consent to participate in this study.

## Author Contributions

A-AK and MA-K: conceptualization and writing—review and editing. A-AK, MA-K, SA, and HH-M: data collection. MA-K: data analysis. MA-K, SA, and HH-M: writing—original draft. A-AK: resources and supervision. All authors have read and approved the manuscript prior to submission.

## Funding

The study was supported by Social Determinants of Health Research Center, Shahid Beheshti University of Medical Sciences under code 20977. The funding body was not in any ways involved in the design of the study and collection, analysis, and interpretation of data and in writing the manuscript.

## Conflict of Interest

The authors declare that the research was conducted in the absence of any commercial or financial relationships that could be construed as a potential conflict of interest.

## Publisher's Note

All claims expressed in this article are solely those of the authors and do not necessarily represent those of their affiliated organizations, or those of the publisher, the editors and the reviewers. Any product that may be evaluated in this article, or claim that may be made by its manufacturer, is not guaranteed or endorsed by the publisher.

## Abbreviations

COVID-19: Coronavirus disease
SD: Standard deviation
95% CI: 95% confidence interval
IQR: Interquartile range
ANOVA: One-way analysis of variance.
